# Novel susceptibility loci for A(H7N9) infection identified by next generation sequencing and functional analysis

**DOI:** 10.1038/s41598-020-68675-y

**Published:** 2020-07-16

**Authors:** Baihui Zhao, Yongkun Chen, Mo Li, Jianfang Zhou, Zheng Teng, Jian Chen, Xue Zhao, Hao Wu, Tian Bai, Shenghua Mao, Fanghao Fang, Wei Chu, Hailiang Huang, Cong Huai, Lu Shen, Wei Zhou, Liangdan Sun, Xiaodong Zheng, Guangxia Cheng, Ye Sun, Dayan Wang, Lin He, Yuelong Shu, Xi Zhang, Shengying Qin

**Affiliations:** 1 Bio-X Institutes, Key Laboratory for the Genetics of Developmental and Neuropsychiatric Disorders, Ministry of Education, Shanghai Jiao Tong University, Shanghai, 200030 China; 2 Shanghai Municipal Center for Disease Control and Prevention, Shanghai, 200036 China; 3 School of Public Health (Shenzhen), Sun Yat-sen University, Shenzhen, 510275 China; 4 National Institute for Viral Disease Control and Prevention, Collaboration Innovation Center for Diagnosis and Treatment of Infectious Diseases, Chinese Center for Disease Control and Prevention, Key Laboratory for Medical Virology, National Health Commission, Beijing, 102206 China; 5 Shanghai Huangpu District Center for Disease Control and Prevention, Shanghai, 200023 China; 6 Analytic and Translational Genetics Unit, Department of Medicine, Massachusetts General Hospital and Harvard Medical School, Boston, MA 02114 USA; 7 Broad Institute of Harvard and MIT, Cambridge, MA 02142 USA; 8 Department of Dermatology, No. 1 Hospital and Key Laboratory of Dermatology, Ministry of Education, Anhui Medical University, Hefei, 230032 China; 9 Jinan Infectious Disease Hospital, Jinan, 250021 China; 10 National Institute for Viral Disease Control and Prevention China CDC, Beijing, 102206 China; 11 Collaborative Innovation Center, Jining Medical University, Jining, 272067 China

**Keywords:** Diagnostic markers, Influenza virus

## Abstract

The A(H7N9) virus strain that emerged in 2013 was associated with a high fatality rate and may become a long-term threat to public health. A(H7N9) disease incidence is disproportionate to viral exposure, suggesting that host genetic factors may significantly influence susceptibility to A(H7N9) infection. Human genome variation in conferring risk for A(H7N9) infection in Chinese populations was identified by a two-stage investigation involving 121 A(H7N9) patients and 187 healthy controls using next generation sequencing followed by functional analysis. As a result, a low frequency variant (rs189256251; *P* = 0.0303, OR = 3.45, 95% CI 1.05–11.35, chi-square test) and three HLA alleles (DQB1*06:01, DQA1*05:05 and C*12:02) were identified in A(H7N9) infected volunteers. In an A549 cell line carrying the rs189256251 variant CT genotype, A(H7N9) infection incidence was elevated 6.665-fold over control cells carrying the CC genotype. Serum levels of interferon alpha were significantly lower in patients with the CT genotype compared to the CC genotype (*P* = 0.01). The study findings of genetic predisposition to A(H7N9) in the Chinese population may be valuable in systematic investigations of A(H7N9) disease etiology.

## Introduction

Since the first reported human infection by the novel avian influenza A virus H7N9 [A(H7N9)] in 2013 more than 1,564 laboratory-confirmed human cases were reported up to 27 May 2018, with a fatality rate of 39.25%. Five large epidemic waves of human infection have been reported^1^. Multiple regionally distinct viral genotypes and expanded geographical range suggest that A(H7N9) virus may become a long-term threat to public health^2^.

The role of host genetic background in determining susceptibility to viral diseases has been studied intensively for several pathogens including human immunodeficiency virus^3^, hepatitis B virus^4^, hepatitis C virus^5^ and respiratory syncytial virus^6^. It is clear that the genetic make-up of the host has a major influence on the response to these pathogens^7^. In 2011 the World Health Organization (WHO) highlighted the importance of host genetic predisposition to infection with influenza virus^8^ and several host genetic variants have been reported to contribute to disease severity and the immune response following influenza virus infection^9,10^.

Because A(H7N9) disease is contracted at rates disproportionate with virus load under circumstances of exposure, it has been suggested that host genetic effects have a major influence on susceptibility to A(H7N9) infection. Studies have investigated single nucleotide polymorphisms (SNPs) as risk factors for predisposition to infection with A(H7N9) virus in influenza patients^11–14^. Other investigations attempted to identify differential gene expression in case-controlled studies^15–17^. For studies involving small numbers of A(H7N9) patients and a possibility that a rare variant might have a moderate to large effect on susceptibility, a next generation sequencing (NGS) strategy might be superior to a genome wide association study (GWAS). Importantly, a stringent GWAS significance threshold and a modest genetic effect size might mask real associations. Therefore, comprehensive analysis using a high-throughput method is required to define additional genetic causes and risk variants for A(H7N9) infection in a relatively large cohort.

Here, we describe a two-stage study involving NGS of Chinese volunteers including 112 A(H7N9) patients and 167 healthy controls followed by functional validation of the effects of genetic variants. We also looked at which human leukocyte antigen (HLA) alleles closely associated with A(H7N9) infection, using HLA target sequencing in a cohort that included 117 A(H7N9) patients and 131 healthy controls. Altogether, our findings may aid systematic understanding of the role of host genetics in A(H7N9) infection.

## Results

### Study design

**Figure 1:**
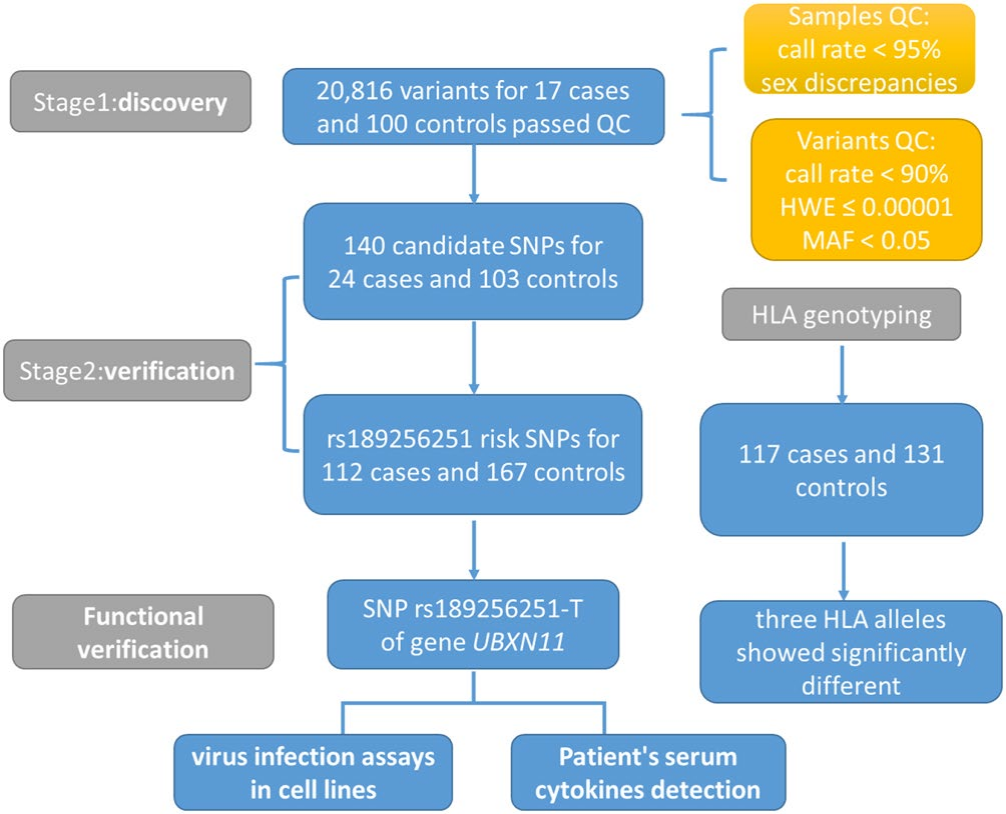
Study design. 121 infected patients and 187 healthy control subjects were enrolled in the study. Stage 1 of the study was designed to identify candidate SNPs and involved whole-exome sequencing (WES) of 17 patients. Stage 2 aimed to verify candidate SNPs and involved 24 patients and 103 controls. SNP rs189256251 was validated in 112 patients vs. 167 control subjects. An in vitro virus infection model was used for functional verification of SNP rs189256251. Serum cytokine levels were measured and compared. In addition, targeted HLA deep sequencing analysis was performed on 117 patients vs. 131 control subjects, identifying three HLA alleles showing association with A(H7N9) infection.

We conducted a two-stage study and the design is shown in Fig. 1. In the discovery stage, whole-exome sequencing (WES) method was used to identify candidate variants in 17 A(H7N9) patients and 100 Chinese Han populations in the 1,000 Genomes Project. In the verification stage, the risk variants were validated in 24 A(H7N9) patients (17 were same with the discovery stage and together with 7 new patients collecting during year 2015–2016) and 103 healthy controls. Then, one chosen risk variant was further replicated in a population including 112 A(H7N9) patients (included the 24 cases in the previous stage) and 167 healthy controls (included the 103 healthy controls in previous stage). HLA gene regions were chosen to do target sequencing in a cohort that included 117 A(H7N9) patients and 131 healthy controls. Clinical and demographic information on the A(H7N9) patients and controls are presented in Supplementary Table S1 online.

### Identification of SNPs

Genomic DNA was collected from 17 A(H7N9) patients. An average of ~ 110.5 million purity-filtered reads were generated for each of the 17 samples. 93% of the target bases in each individual were covered by at least 10 independent sequence reads, with an average sequence depth of 76.2× in each sample (see Supplementary  Table S2 online). These data were compared with data from 100 Southern Han Chinese individuals in the 1,000 Genomes phase 1 release. A total of 29,484 markers from 17 patients and 100 control subjects passed the quality filter. After genotyping pruning (removing minor allele frequency (MAF) > 0.05 variants) a total of 20,816 variants remained for association analysis. We used two approaches to select a subset of SNPs for further analysis. In one approach, we identified novel SNPs (not reported in the 1,000 Genomes database) that were detected in WES data of at least 80% of patients. In the other approach, we used the Pearson chi-squared test to evaluate the significance of allele frequency comparisons between cases and controls and to generate P-values. In total, 140 missense or loss of function (LOF) mutations that were novel or met the significance threshold (*P* < 1 × 10^–4^) were defined as candidate SNPs for follow-up study (see Supplementary Table S3 online).

### Verification of risk associated SNP rs189256251

The 140 candidate SNPs described above plus 6 previously reported SNPs (see Supplementary Table S3 online) were verified in analysis of 24 A(H7N9) patients and 103 healthy controls. Primers used in verification are shown in Supplementary Table S4 online. After quality control, 120 candidate SNPs and 6 known SNPs from 22 A(H7N9) patients and 103 healthy controls were tested by chi-square and 17 SNPs at 11 loci were identified as risk markers with an unadjusted *P* value below 0.05 (Table 1). Following Bonferroni correction only rs189256251 and rs116914994 showed significant association with A(H7N9) infection (*P* = 0.00803, *P* = 0.0338, respectively). Significant association of missense mutation rs189256251 in the UBX Domain Protein 11 (*UBXN11*) gene was still evident following logistic regression analysis (*P* = 0.00089, OR = 24.92, 95% CI 3.742–165.9) but none of the 6 known SNPs showed an association with A(H7N9) infection at this stage of analysis.

**Table 1:** Verification of significant SNP association.

Chr	SNP	Locus	Gene	Unadjusted		Adjusted	
				*P*	OR	Bonferroni	FDR_BH
1	rs189256251	1p36.1	*UBXN11*	0.000149	13.08	0.00803	0.00803
14	rs116914994	14q24.3	*LTBP2*	0.000626	4.367	0.0338	0.0169
9	rs57657464	3q13.2	*CD200R1*	0.00549	4.489	0.296	0.0771
3	rs6804162	3q13.1	*GUCA1C*	0.00572	2.533	0.308	0.0771
2	rs61732371	2q36.2	*FAM124B*	0.0119	7.463	0.643	0.0946
6	rs78446650	6p12.1	*MLIP*	0.0119	7.463	0.643	0.0946
3	rs2259102	3q29	*MUC4*	0.0123	2.387	0.66626	0.0946
3	rs2177336	3q29	*MUC4*	0.0162	2.279	0.872	0.109
8	rs79493663	8q24.3	*MROH1*	0.0248	9.762	1	0.143
7	rs10271996	7p14.3	*PRR15*	0.0315	0.478	1	0.143
3	rs1104760	3q29	*MUC4*	0.0353	2.073	1	0.143
3	rs2293232	3q29	*MUC4*	0.0353	2.073	1	0.143
3	rs882605	3q29	*MUC4*	0.0353	2.073	1	0.143
9	rs2275156	9q34.3	*MAMDC4*	0.0425	0.243	1	0.143
3	rs2688513	3q29	*MUC4*	0.0425	2.017	1	0.143
3	rs1106502	3q29	*MUC4*	0.0425	2.017	1	0.143
13	rs2282406	13q12.1	*AMER2*	0.0479	2.595	1	0.152

Chr, chromosome; SNP, single nucleotide polymorphisms; OR, odds ratio; P value is for the chi-squared test, FDR, false discovery rate.

Further validation of SNP rs189256251 in 112 A(H7N9) patients and 167 healthy controls continued to indicate significant association (*P* = 0.0303, OR = 3.45, 95% CI 1.05–11.35, chi-square test). All patients or controls with the rs189256251 (C>T) mutation were detected again by Sanger sequencing and all results showed the mutation occurring in both alleles.

### Pathogenic potential of rs189256251

Ingenuity pathway analysis (IPA) showed that *UBXN11* interacts with *EIF4E* and *RNF213,* homologs of which (*EIF4B* and *RNF5*) were previously reported to be associated with influenza virus infection through modulation of related signaling pathways^18,19^. The Genecard database shows *UBXN11* encoding a member of the ubiquitin binding factor X (UBX) family and having a divergent C-terminal UBX domain. The rs189256251 (a missense mutation NP_892120.2:p.Arg400His) SNP introduces a striking change in the 3-D structure of the C-terminal active domain of UBXN11 (see Supplementary Fig. S1 online). In addition, it has been reported that another member of the UBX family, *UBXN1*, plays an important role during influenza virus infection by regulating the host antiviral immune response^20^. We therefore performed further functional verification studies on SNP rs189256251.

### Rs189256251 CT genotype related to infection

**Figure 2:**
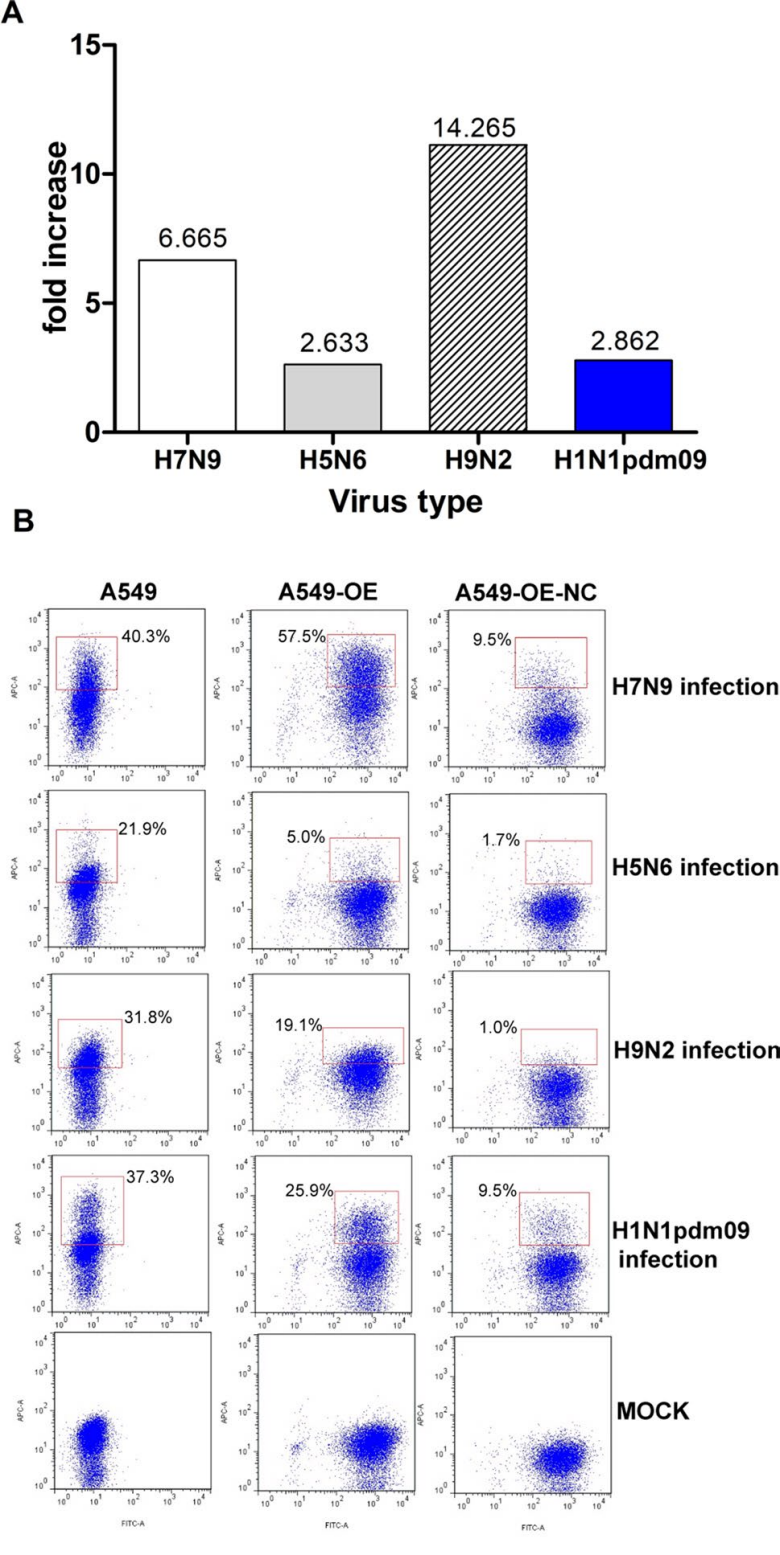
Functional verification of rs189256251 genotype CT. (**A**) Fold increase of infection calculated based on the mean A549 cell line OE: OE-NC % infection. Values were 6.665, 2.633, 14.265 and 2.862 fold increase for A(H7N9), A(H5N6), A(H9N2) and pandemic H1N1 2009 virus infection, respectively. (**B**) Representative flow cytometry results of virus A(H7N9), A(H5N6), A(H9N2) and pandemic H1N1 2009 infections of control A549 cells, cells overexpressing the CT genotype of rs189256251 (OE) and negative control (OE-NC) cell lines carrying empty viral vector. A549 cells mock infected with influenza A are also shown as a control. All flow cytometry assay results are presented in one quadrant. The X-axis represents EGFP (green) fluorescence intensity. The Y-axis represents ACP (red) fluorescence intensity. The red square and number show infected cell.

The A549 OE and OE-NC cell lines harbor *UBXN11* with CT genotype and empty lentivirus vectors, respectively. The transcription level of *UBXN11* (CT) in OE cells was 5.565 fold increased compared to OE-NC cells. As shown in Fig. 2A and Supplementary Table S5 online, OE cells were 6.665-fold, 2.633-fold, 14.265-fold and 2.862-fold more infected with A(H7N9), A(H5N6), A(H9N2) and pandemic H1N1 2009 viruses, respectively, than OE-NC cells. Representative flow cytometry results for influenza virus infection assays are shown in Fig. 2B.

### Rs189256251 CT genotype associated with reduced serum interferon alpha

**Figure 3:**
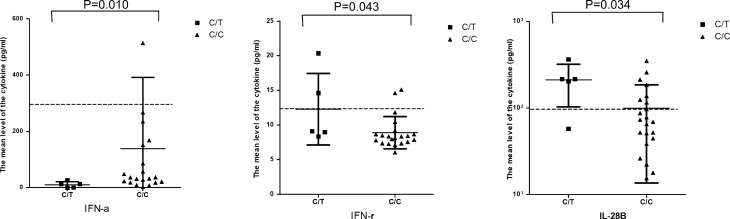
Patient serum chemokine/cytokine levels Serum sample acceptance criteria were (1) the first serum obtainable from A(H7N9) patients and (2) an interval between disease onset and sample collection not exceeding 12 days. IFN-γ levels were determined using the cytokine bead array (CBA) and flow cytometry (FACSAria BD Biosciences, USA). IFN-α and IL-28B were determined using sandwich commercial enzyme-linked immunosorbent assay kits (LifeSpan, USA). Significantly decreased serum levels of interferon alpha (type I interferon, *P* = 0.01) and increased levels of interferon gamma (type III interferon, *P* = 0.043) and IL-28B (type II interferon, *P* = 0.034) were associated with the CT genotype of rs189256251.

Serum was collected from five patients with the CT genotype and twenty-one patients with the CC genotype. Cytokine levels were determined and compared using the Mann–Whitney U test (see Supplementary Table S6 online). Significantly decreased levels of interferon alpha (type I interferon, *P* = 0.01) and increased levels of interferon gamma (type III interferon, *P* = 0.043) and IL-28B (type II interferon, *P* = 0.034) were found in patients with the CT genotype compared to those of genotype CC (Fig. 3).

### Classical HLA types linked to A(H7N9) susceptibility

Six classical HLA loci were target-sequenced in one cohort. The primers used are shown in Supplementary Table S4 online. The overall average sequencing depth was above 200× with Q30 more than 80%. The sequencing depth was > 140×  for > 90% of samples. *P*-values were calculated using the Fisher exact test. We identified three HLA alleles differing between A(H7N9) patients and controls: HLA-DQB1*06:01 (*P* = 0.022, OR = 0.121, CI 0.015–0.960), HLA-DQA1*05:05 (*P* = 0.023, OR = 0.197, CI 0.043–0.897) and HLA-C*12:02 (*P* = 0.039, OR = 0.217, CI 0.047–1.002). These three HLA alleles also showed significant differences compared with 10,689 previously published control subjects with effects in the same direction (Table 2).

**Table 2:** HLA allele association.

HLA allele	Present study					Previous study			
	Case	Control	P	OR	95% CI	Control	P	OR	95% CI
DQA1*05:01	3	17	0.0048	0.187	0.054–0.647	605	0.220	0.446	0.091–1.326
A*02:06	15	4	0.0082	4.418	1.445–13.51	1,213	0.732	1.139	0.625–1.928
B*13:02	16	6	0.016	3.131	1.204–8.142	1,520	0.973	0.959	0.537–1.598
DQB1*06:01	1	9	0.022	0.121	0.015–0.960	2,005	4.38E–04	0.065	0.0016–0.367
DQA1*05:05	2	11	0.023	0.197	0.043–0.897	1,322	1.17E–03	0.131	0.0157–0.479
DRB1*13:01	2	11	0.023	0.197	0.043–0.897	320	0.593	0.567	0.068–2.089
DRB1*12:02	38	64	0.026	0.600	0.383–0.938	1,531	2.04E–07	2.513	1.720–3.587
C*12:02	2	10	0.039	0.217	0.047–1.002	778	0.0361	0.228	0.027–0.837
DRB1*15:01	29	50	0.049	0.600	0.365–0.985	2,723	0.953	0.970	0.632–1.438

HLA, human leukocyte antigen; OR, odds ratio; CI, confidence interval; P value is for the Fisher test.

## Discussion

Multiple studies have demonstrated that host susceptibility to influenza virus is determined by host genetics^21^. Most studies validated previously reported variants and only two were designed to search for new gene variants associated with disease susceptibility. Chen et al*.* reported functional variants in Galectin 1 affecting susceptibility to influenza A(H7N9) using a GWAS approach^22^. However, given the small number of A(H7N9) cases involved in the study, GWAS likely not the optimal approach^21^. A WES approach yielded 21 genes related to A(H7N9) infection^13^, but here again the sample size was relatively small.

In this study, we performed a two-stage study of host genetic predisposition using next generation sequencing based techniques to analyze a Chinese population that included 121 laboratory-confirmed A(H7N9) patients. We identified one low frequency SNP and three HLA alleles showing significant association with A(H7N9) infection. For all 112 patients, the allele frequency of the T allele of SNP rs18925625 was 4.02% (9/224), significantly higher than 0.5% (1/199) in the Southern Han Chinese population in the 1,000 Genomes phase 1 release, and significantly higher than 0.77% (67/8,622) in the East Asian population in the ExAC database. This was followed by functional verification of the SNP in an in vitro cell infection model. These studies showed that overexpression of UBXN11 (CT) rendered cells significantly more susceptible to A(H7N9) virus infection. The cells were also more susceptible to infection by A(H9N2), A(H5N6) and pandemic H1N1 2009 viruses. We also found reduced serum levels of interferon alpha in patients carrying the CT genotype compared to CC genotype patients. It is well known that interferon alpha is linked to innate immunity, which is critical for eliminating virus soon after infection^23^. *UBXN11* therefore may modulate host susceptibility to the A(H7N9) virus through down regulation of innate immunity. Indeed, a previous study reported that *UBXN1*, another member of the UBX-domain containing family, strongly inhibited the RNA virus-induced type I interferon response^20^.

The HLA locus is involved in regulation of viral immunity. A previous study reported preferential binding of HLA alleles to conserved regions of viral proteomes and suggested that this preference may provide improved clearance of infection in different viral infections^24^. Other studies have suggested that HLA-DR levels in CD14^+^ cells may be a biomarker predicting H7N9 disease progression^25^ and reported divergent differentiation pathways of CD38^+^ HLA-DR^+^ CD8^+^ T cells during fatal H7N9 disease^26^. These results support the argument that the HLA alleles identified in the current study may be useful markers for assessing risk of A(H7N9) infection.

There were also some limitations of this study. First, compared with the sequencing analysis of other diseases, our sample size is not large. But considering the overall number of A(H7N9) disease our sample is considered sufficient. Cytokines were measured in serum from only five patients with the CT genotype and 21 patients with the CC genotype. Future studies should explore in larger study populations that have a bigger sample size of cases. In addition, HLA gene alleles are highly polymorphic, and some alleles have low frequencies. Testing on a small sample size will cause some errors, which also needs to be verified on a larger sample size.

In summary, we identified several genetic variants associated with A(H7N9) infection. The two-stage study design improved confidence in the results from a relatively small cohort, beneficial when the disease frequency is low. All of the identified variants are linked to antiviral immunity, highlighting the importance of innate immunity in genetic predisposition to A(H7N9) infection. Fine mapping and functional studies of the identified loci may provide further insights into the etiology of A(H7N9) disease.

## Materials and methods

### Study subjects

All subjects were recruited and anonymized by staff of the Shanghai Municipal Center for Disease Control and Prevention (SCDC) or the Chinese National Influenza Center (CNIC) in the course of an epidemiological survey. A total of 121 A(H7N9) patients and 187 healthy controls were recruited. A(H7N9) infection was in each case confirmed by real-time reverse-transcription polymerase chain reaction and/or virus culture. Heavily exposed poultry workers or close contacts were recruited as healthy controls. Venous blood samples and nasal swabs were collected from subjects. Genomic DNA was extracted from venous blood using a Qiagen Blood Kit (Qiagen, Germany) according to the manufacturer’s instructions.

All procedures performed in studies involving human participants were in accordance with the ethical standards of Bio-X Institutes of Shanghai Jiao Tong University and National Institute for Viral Disease Control and Prevention (approval number IVDC2014-020) and with the 1964 Helsinki declaration and its later amendments or comparable ethical standards. Informed consent was obtained from all individual participants included in the study.

### WES and data quality control

17 A(H7N9) patients’ DNA exomes were ‘captured’ using Illumina TruSeq Exome enrichment technology (Illumina, USA). Libraries were indexed, pooled and sequenced on an Illumina HiSeq2000 platform (paired-end, 100-bp reads, 5 or 6 libraries per lane). Raw sequencing reads were aligned to the reference genome hg19 using the Burrows-Wheeler Aligner (BWA, version 0.7.12). Duplicate reads and reads mapping to more than one location were excluded. The Genome Analysis Toolkit (GATK, version 3.5) was used for SNP calling. All SNPs were filtered using the GATK variant filtration module with a hard filter setting for initial filtering. Variants were annotated as a stop gain, stop loss, synonymous, nonsynonymous or splicing mutation using ANNOVAR^27^.

A uniform quality control protocol was used to filter the samples and SNPs^28^. Samples that failed to reach a genotype call rate of 95% and those with sex discrepancies were excluded. SNPs were excluded based on (i) not mapping on an autosomal chromosome, (ii) displaying a low call rate (< 90%) in all subjects and (iii) violating Hardy–Weinberg equilibrium (HWE) (*P* ≤ 0.00001) in the controls. We used two methods to select SNP sites for subsequent verification. One method was to identify novel, potentially harmful mutations that are not reported in the 1,000 Genomes database (novel SNPs) but were detected in at least 80% of patients. The other method was to compare allele frequencies between cases and controls and generate a P-value using the Pearson chi-squared test. Due to the relatively small sample size in the WES stage we set *P* < 1 × 10^–4^ as the significance threshold in order to select more SNPs. We then focused on nonsynonymous and LOF (including stop gain, stop loss and splicing) mutations in the verification stage.

### Validation of candidate SNPs

In order to validate discoveries, candidate SNPs were detected by Agena iPLEX MassARRAY assay, multiplex SNaPshot genotyping or Sanger sequencing to achieve highest confidence results in a verification stage involving 24 A(H7N9) patients (including 17 of the same patients studied in the discovery stage and 7 new patients) and 103 healthy controls. Moreover, 6 known SNPs associated with influenza infection based on previous studies^16,29–32^ were evaluated. For quality control of genotyping, blinded duplicate samples from two subjects and two negative control (water) samples were included in each method. Samples that failed to reach a call rate of 90% and SNPs displaying a call rate below 95% or HWE *P* ≤ 0.00001 were excluded. Logistic regression was performed to subtract effects of age and sex on results. Lastly, the SNP with the most significant association with A(H7N9) disease was confirmed in a cohort including 112 A(H7N9) patients and 167 healthy controls. At this stage, our samples are completely covered the previous stage.

### In silico analysis

Interactions between the *UBXN11* gene and other genes were evaluated using commercial IPA software (Qiagen, Redwood City, CA, USA) based on pathways previously defined in the literature. The gene was characterized based on Genecard database (https://www.genecards.org/) and PubMed search results. Because *UBXN11* encodes a 520 amino acid protein with a divergent C-terminal UBX domain we modeled the 3-D structure of the C-terminal active domain of *UBXN11* with and without the rs189256251-T mutation using Swiss-Model online software (https://www.swissmodel.expasy.org/)^33^. Figures of the 3-D structure were generated by Swiss-PdbViewer software (version 4.1.0, https://www.expasy.org/spdbv).

### Cell lines and virus infection assays

Adenocarcinoma derived human alveolar basal epithelial (A549) cells were used in functional validation assays of the CT genotype of rs189256251. A549 cells were purchased from the cell bank of the Chinese Academy of Science. Before assay, the genotype of rs189256251 in the original A549 cell were sequenced by Sanger sequencing and the result showed rs189256251 was the CC genotype. The stable A549 overexpression cell line (A549 OE) and negative control (OE-NC) were constructed using lentivirus vectors with or without the target CT genotype of rs189256251 (Shanghai Genechem Co. Ltd). The transcription levels of *UBXN11* in the OE and OE-NC cell lines were measured by real time PCR in triplicate assays and values calculated relative to a *GAPDH* internal control. Both OE and OE-NC cell lines also expressed EGFP.

Four types of influenza A viruses were included in assays: WHO recommended A(H7N9) avian influenza virus vaccine strain (NIBRG267), low pathogenicity A(H5N6) avian influenza virus (GDH5N6RETR), low pathogenicity A(H9N2) avian influenza virus (A/HK/2009(H9N2)) and pandemic H1N1 2009 influenza virus (A/Shanghai HP/SWL13439/2016(H1)). The tissue culture infective dose 50 (TCID_50_) for each virus type was determined using Madin-Darby canine kidney (MDCK) cells. A549, OE and OE-NC cells were inoculated with virus at a multiplicity of infection (M.O.I) of 1. The final concentration of TPCK-treated trypsin (Sigma, USA) used during infection was 0.5 g/ml. Cells were harvested 12 h post infection using 0.25% EDTA-trypsin. Cells were washed with PBS, fixed and permeabilized using Cytofix/Cytoperm solution (BD, USA). The mouse monoclonal antibody FR-1217 (WHO) against influenza A virus matrix protein was used to detect intracellular virus, followed by APC labeled goat anti-mouse IgG (Biolegend, USA) secondary antibody. Infected cells were detected by flow cytometry (FACSAria III, BD Biosciences, USA). Infection assays for A(H7N9) and A(H5N6) viruses were performed three times in parallel, while those for A(H9N2) and pandemic H1N1 2009 viruses were performed twice. A549 cell line without influenza virus infection was included in each infection assay to establish baseline fluorescence levels in flow cytometry. The fold-increase in infection was calculated using the OE to OE-NC cell mean ratio of percent infection.

### Serum cytokine levels

For serum cytokine detection, sample acceptance criteria were first serum sample collected from A(H7N9) patient and interval between disease onset and sample collection of less than 12 days. Serum levels of IL-1β, IL-2, IL-4, IL-5, IL-6, IL-8, IL-10, IL-12P70, IL-13, IL-17A, IL-21, IL-23, MIP-1a, MIP-1β, MCP-1, MIG, IP-10, RANTES, and IFN-γ were evaluated using the cytokine bead array (CBA) and the Human Th1/Th2 Cytokine CBA kit (BD Biosciences, San Jose, CA, USA) in a flow cytometer (FACSAria BD Biosciences, USA) as previously described^34^. Data were analyzed using BD CBA analysis software. The individual cytokine concentrations in each test sample were determined using a standard curve. Cytokine levels were measured in duplicate, averaged and compared to a standard curve. IFN-α and IL-28B were detected by sandwich commercial enzyme-linked immunosorbent assay kits (LifeSpan, USA). Data analysis was performed according to kit manufacturer’s instructions.

### HLA typing

Six classical HLA loci (*HLA-A, HLA-B, HLA-C, HLA-DQA1, HLA-DQB1* and *HLA-DRB1*) were chosen for targeted deep sequencing in a cohort that included 117 A(H7N9) patients and 131 healthy controls. HLA sequences were amplified by multiplex PCR and sequenced in PE 250 sequencing mode on the Illumina MiSeq sequencer according to manufacturer’s instructions. Low quality reads and those contaminated with adaptor sequences were filtered out to yield clean reads. HLA allele typing was performed using Omixon Target software (version 1.8.1). The HLA genotyping data from 117 A(H7N9) patients were also compared to previously published data from 10,689 Han Chinese control subjects^35^.

### Statistical analysis

Plink software (version 1.9) was used to evaluate association between each variant and A(H7N9) infection risk. Male *vs.* female ratio and age distribution were compared by Mann–Whitney U test and two-tailed Student’s t-test, where appropriate, using SPSS software (version 19.0.0). Fisher's exact test was used to compare HLA frequency between infected and healthy controls. *P* < 0.05 was considered statistically significant.

## Supplementary information

Supplementary Table S1.

Supplementary Table S2.

Supplementary Table S3.

Supplementary Table S4.

Supplementary Table S5.

Supplementary Table S6.

Supplementary Figure S1.

## Data Availability

The samples’ sequences generated during the current study are available in SRA repository: SRR7263353-SRR7263369.
