# Supplementary material for: Novel susceptibility loci for A(H7N9) infection identified by next generation sequencing and functional analysis
Source: Sci Rep. 2020 Jul 16;10:11768. doi: 10.1038/s41598-020-68675-y (PMC7366728; doi:10.1038/s41598-020-68675-y)
Supplement: Supplementary file 7 — Supplementary Figure S1. [file 41598_2020_68675_MOESM7_ESM.docx]

**Title:** Novel susceptibility loci for A(H7N9) infection identified by next generation sequencing and functional analysis

Baihui Zhao,Yongkun Chen, Mo Li,Jianfang Zhou, Zheng Teng, Jian Chen, Xue Zhao, Hao Wu, Tian Bai, Shenghua Mao, Fanghao Fang, Wei Chu, Hailiang Huang, Cong Huai, Lu Shen, Wei Zhou, Liangdan Sun, Xiaodong Zheng, Guangxia Cheng, Ye Sun, Dayan Wang, Lin He, Yuelong Shu, Xi Zhang and Shengying Qin


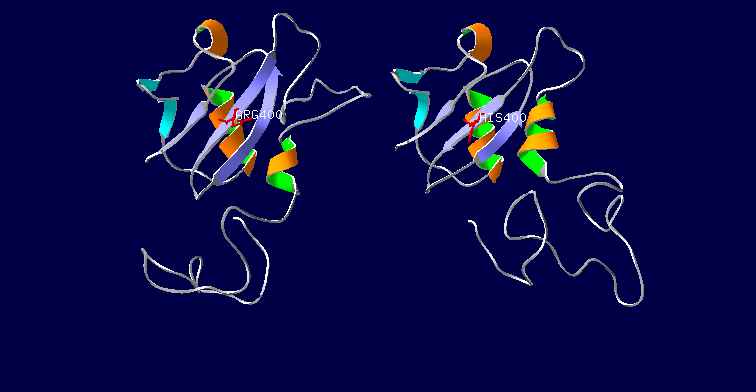


**Supplementary Fig. S1. The striking change C-terminal UBX domain of UBXN11 induced by rs189256251.** The left represents wild type, and the right represents with rs189256251 (NP_892120.2:p.Arg400His) mutation 3-D structure of UBX domain. Figures of the 3-D structure were generated by Swiss-PdbViewer software (version 4.1.0, https://www.expasy.org/spdbv). In the wild type, position 400 falls on a β-sheet consisting of 6 amino acids, while rs189256251 make the original β-sheet 6 amino acids into 3. The other β-sheet are also shorter.
